# Unnatural Amino Acid Photo-Crosslinking Sheds Light on Gating of the Mechanosensitive Ion Channel OSCA1.2

**DOI:** 10.3390/ijms26157121

**Published:** 2025-07-23

**Authors:** Scarleth Duran-Morales, Rachel Reyes-Lizana, German Fernández, Macarena Loncon-Pavez, Yorley Duarte, Valeria Marquez-Miranda, Ignacio Diaz-Franulic

**Affiliations:** Center for Bioinformatics and Integrative Biology, Universidad Andres Bello, Santiago 8370146, Chile; mduran.scarleth@gmail.com (S.D.-M.); rachel.reyes2b@gmail.com (R.R.-L.); germanfv@gmail.com (G.F.); maca.blp16@gmail.com (M.L.-P.); yorley.duarte@unab.cl (Y.D.); valeria.marquez@unab.cl (V.M.-M.)

**Keywords:** mechanosensing, OSCA1.2, unnatural amino acids (UAAs), ion channel gating

## Abstract

Mechanosensitive ion channels such as OSCA1.2 enable cells to sense and respond to mechanical forces by translating membrane tension into ionic flux. While lipid rearrangement in the inter-subunit cleft has been proposed as a key activation mechanism, the contributions of other domains to OSCA gating remain unresolved. Here, we combined the genetic encoding of the photoactivatable crosslinker p-benzoyl-L-phenylalanine (BzF) with functional Ca^2+^ imaging and molecular dynamics simulations to dissect the roles of specific residues in OSCA1.2 gating. Targeted UV-induced crosslinking at positions F22, H236, and R343 locked the channel in a non-conducting state, indicating their functional relevance. Structural analysis revealed that these residues are strategically positioned: F22 interacts with lipids near the activation gate, H236 lines the lipid-filled cavity, and R343 forms cross-subunit contacts. Together, these results support a model in which mechanical gating involves a distributed network of residues across multiple channel regions, allosterically converging on the activation gate. This study expands our understanding of mechanotransduction by revealing how distant structural elements contribute to force sensing in OSCA channels.

## 1. Introduction

Mechanosensitive ion channels are essential molecular components that allow cells to sense and respond to mechanical stimuli, mediating critical physiological processes such as osmoregulation, hearing, and touch [1,2]. Among these, OSCA channels (also known as TMEM63 in animals) are widely expressed in plant tissues, with OSCA1.2 primarily localized in root cells where they help to modulate turgor pressure and osmotic balance in response to external stress [3,4,5]. OSCA1.2 is a hyperosmolality-gated, calcium-permeable mechanosensitive channel found in plants that triggers calcium-dependent signaling to increased soil salinity [6]. Its mammalian orthologs have been implicated in various physiological and pathological processes, including cell differentiation [7], lung surfactant secretion [8], axonal hypomyelination [9,10], and neurodegenerative diseases such as amyotrophic lateral sclerosis (ALS) [11]. OSCA1.2 forms a homodimeric, mechanically activated ion channel in which each subunit comprises 11 transmembrane helices (TM0–10) and an intracellular domain (ICD) that serves as the sole dimerization interface [12,13]. The core ion-conducting pore is located within each monomer and is primarily lined by TM3–7, while TM0 is a peripheral helix embedded in the lipid bilayer. The two subunits are separated by an 8–10 Å cleft filled with lipid molecules, which are thought to modulate gating via the “force-from-lipid” mechanism [6,14]. The ICD, composed of β-sheets and several α-helices—including an amphipathic helix and a membrane-associated Beam-Like Domain (BLD)—provides the structural basis for dimer formation and potential mechanical signal transduction, echoing structural features of the TMEM16 family despite low sequence identity [14]. The activation gate of OSCA1.2 is in the middle of the pore and involves hydrophobic constriction by pore-lining residues, notably Y519 in TM6, which is hypothesized to undergo conformational rearrangement during gating transitions. This tyrosine, positioned near the extracellular end of the conduction path, is believed to form part of a steric gate that responds to membrane tension. Additionally, the TM6a–TM7 intracellular linker, shown in related isoforms (e.g., OSCA2.3) to constrict the intracellular pore, is a mobile structural element that may act as a secondary gate or inactivation domain. Its flexibility and positioning are strongly coupled to the BLD, suggesting a coordinated movement that could relay mechanical stimuli from the membrane to the pore [13,14]. Cryo-EM structures reveal lipid-like densities in the inter-subunit cleft and near a fenestration that exposes the pore to membrane lipids. These findings support a model where lipid displacement from this fenestration facilitates pore opening, effectively coupling membrane deformation to structural rearrangements within the channel [12,14]. However, the precise molecular sequence of events linking lipid displacement, ICD rearrangement, and pore opening remains unclear. Addressing this question is essential to fully understand the mechanogating mechanism of OSCA channels and their broader role in mechanotransduction. Unnatural amino acids (UAAs) have emerged as a powerful tool for studying membrane proteins, overcoming limitations in residue targeting and temporal control [15]. Genetically encoded photoactivatable crosslinkers, such as p-benzoyl-L-phenylalanine (BzF), enable the UV-light-driven immobilization of specific residues, allowing for the precise investigation of structural roles during gating transitions [16,17,18,19]. In this study, we used BzF-mediated crosslinking to identify the molecular drivers of mechanogating on the OSCA1.2 channel. Six residues located at the ICD, the lipid–channel peripheral interface, and near the conduction pathway were targeted to assess their roles in channel gating. We used calcium imaging as a proxy for channel activation levels and performed molecular dynamics simulations and dynamic cross-correlation analysis to gain insight into the molecular consequences of crosslinking. We found that restricting the movement of F22, located at the peripheral membrane interface, H236 at the ICD, and R343 at the inter-subunit interface apparently trapped the channel in a closed state. Molecular simulations revealed that each of these residues interact differently with the channel gating machinery, suggesting that multiple structural inputs converge on the activation gate to regulate mechanogating.

## 2. Results

### 2.1. Conformational Dynamics at F22, H236, and R343 Is Required for OSCA1.2 Gating

**Figure 1:**
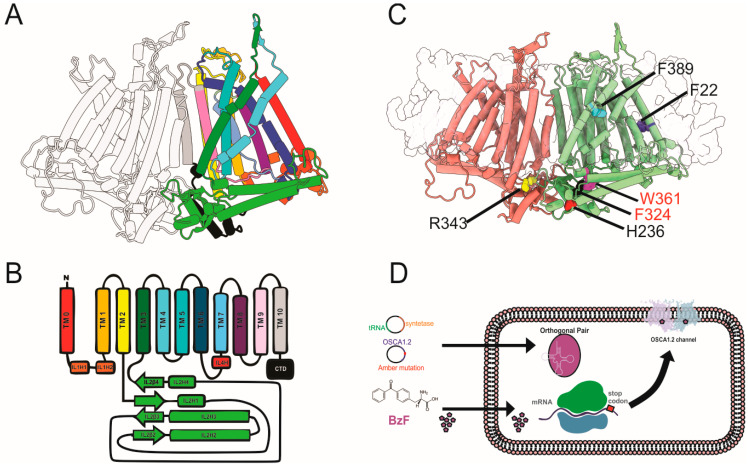
Structural organization of OSCA1.2 channel and location of BzF-substituted residues. (**A**) Ribbon diagram of the transmembrane protein complex showing one monomer highlighted in color and the remaining subunit in gray. Individual transmembrane helices (TMs) are colored distinctly to illustrate their arrangement within the membrane according to the labels in (**B**). Intracellular domain is shown in light green. (**B**) Schematic topology model of a single monomer, displaying the arrangement of transmembrane helices (TM0–TM10), N-terminal domain (NTD), and C-terminal domain (CTD). Intracellular helices (ILH1–ILH4) and intracellular loops (IL1–IL4) are shown below the membrane-spanning region. (**C**) OSCA1.2 channel in a dimeric assemble highlighting residues substituted with BzF for crosslinking assays (see Figure 2). Residues F22, H236, F324, R343, W361, and F389 (green subunit) are shown in van der Waals representation. (**D**) Schematic of the genetic code expansion strategy used for site-specific incorporation of BzF into OSCA1.2. The amber stop codon (TAG) is introduced at selected sites in the OSCA1.2 gene. Co-expression of an orthogonal tRNA/synthetase pair enables BzF incorporation during translation, allowing UV-induced crosslinking of proximal residues in the protein.

**Figure 2:**
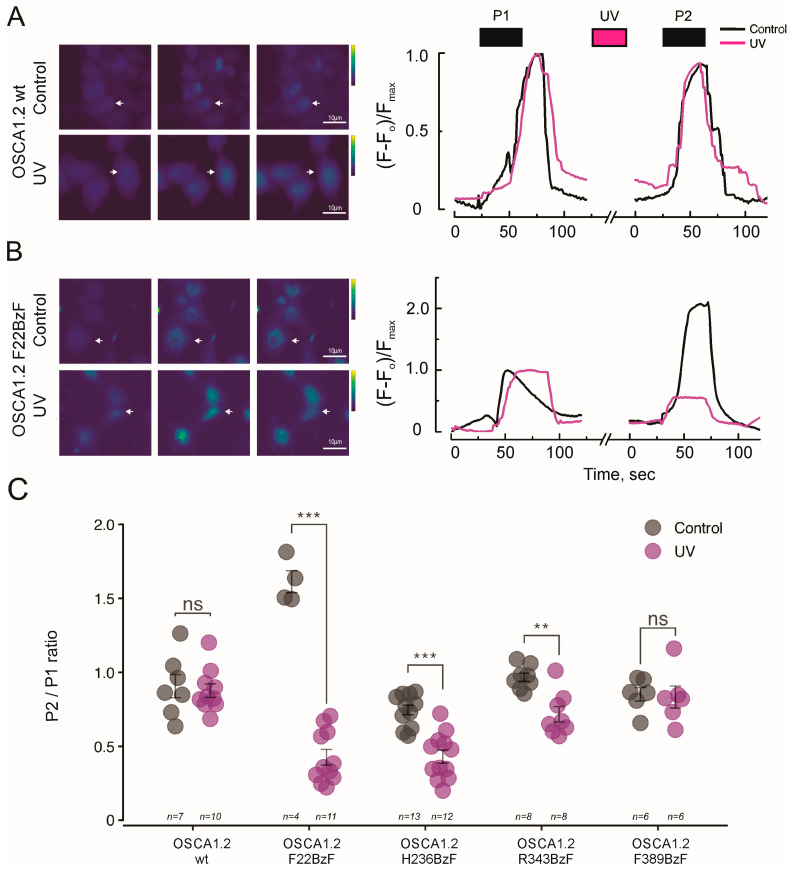
UV-induced photo-crosslinking at specific residues disrupts OSCA1.2 channel opening. Representative pseudocoloring of HEK293T *Piezo1*^−/−^ cells expressing (**A**) OSCA1.2-wild-type and (**B**) OSCA1.2-F22BzF in control (upper panels) and UV-treated conditions (lower panels). Images show three time points: baseline (C), after the first hyperosmotic stimulus (P1), and after the second stimulus (P2), which followed a 30–40 s UV pulse. White arrows indicate representative cells used for quantification. Scale bars: 10 μm. Line plots display fluorescence responses over time; black and purple bars above indicate timing of hyperosmotic pulses and UV exposure, respectively. (**C**) Quantification of calcium response ratios (P2/P1) for five OSCA1.2 constructs under control (gray) and UV-treated (purple) conditions. Each dot represents one n (average of 10 cells), and sample sizes are shown below each group. UV exposure significantly reduced P2/P1 ratios in F22BzF, H236BzF, and R343BzF mutants, but not in wild-type or F389BzF channels. Data represent mean ± SEM. Unpaired two-tailed *t*-tests were used for statistical comparisons. ** *p* < 0.01; *** *p* < 0.001; ns, not significant.

To identify the critical residues involved in mechanogating of OSCA1.2, we used calcium imaging in HEK293 *Piezo1*^−/−^ cells to screen six mutant channels, each containing a targeted residue replaced with the photoactivatable unnatural amino acid BzF [20]. Cells were transfected with each OSCA1.2 construct were loaded with Fluo-4 AM calcium dye and exposed to a solution containing 600 mM of sodium chloride in order to raise the medium osmolarity and trigger channel opening [4]. Changes in intracellular calcium levels were recorded as a proxy for channel activation. As illustrated in Figure 1A, the OSCA1.2 dimer is composed of eleven transmembrane helices and several intracellular segments [6,14], with one subunit shown in rainbow coloration and the second in gray for clarity. A schematic topology diagram is provided in Figure 1B, highlighting the transmembrane helices (TM0–TM10), intracellular loops (ILHs), and the cytosolic C-terminal domain (CTD), which together define the domain architecture of the channel. We targeted residues positioned across structurally and functionally distinct regions, as shown in Figure 1C. These include F22, which is positioned in TM0 at the membrane–protein interface, close to TM6 and the proposed activation gate; H236 is found in the intracellular loop IL4, near the cytoplasmic domain; and R343 is positioned in the TM4–TM5 loop, facing inward toward the transmembrane bundle and possibly involved in intra-subunit interaction. Residues F324 and W361 are located at the channel internal aspects, and F389 is located within TM3 at the membrane core. Each residue was substituted with the photoactivatable non-canonical amino acid BzF by inserting an amber stop codon in place of the original codon in the *osca1.2* gene sequence. The plasmid was then co-transfected with the orthogonal tRNA/synthetase pair, as depicted in Figure 1D. Except for the red-labeled variants in Figure 1C, all OSCA1.2-BzF mutants were successfully expressed and functional in calcium fluorescence assays, supporting their structural integration into the channel complex. To evaluate the functional impact of site-specific photo-crosslinking on OSCA1.2 gating, we compared calcium responses in BzF-substituted mutants subjected to UV illumination between two consecutive hyperosmotic stimuli (P1 and P2). Figure 2A shows Fluo-4 loaded HEK293 *Piezo1*^−/−^ cells expressing wild-type OSCA1.2 channels during control, pre-stimulus condition (C), during the first hyperosmotic test pulse (P1) and during the second hyperosmotic test pulse (P2), for both control (no UV exposure between P1 and P2) and UV exposure conditions. Control and UV-exposed cells exhibited consistent calcium influx across both pulses, indicating that UV exposure had no detectable effect on native channel activity (Figure 2A). Figure 2B shows HEK293 *Piezo1*^−/−^ cells expressing OSCA1.2-F22BzF channels loaded with Fluo-4. This variant exhibited significant potentiation during P2 under control conditions, a trend observed across different experiments (see Section 3). However, the UV-treated F22BzF mutant displayed a significant reduction in calcium influx during P2 (Figure 2B), suggesting that crosslinking at these positions interferes with conformational changes required for channel reopening. The quantification of P2/P1 response ratios confirmed a statistically significant impairment in UV-treated F22BzF, H236BzF, and R343 constructs, whereas F389BzF and wild-type channels showed no significant differences between pulses (Figure 2C and Supplementary Figure S1A–C). These findings identify F22, H236, and R343 as structurally dynamic residues essential for channel activation, and demonstrate the utility of BzF-mediated crosslinking in probing gating mechanisms. As a control, non-transfected HEK293 *Piezo1*^−/−^ cells did not exhibit calcium responses to hyperosmotic stimulation (Supplementary Figure S1D), confirming the absence of endogenous mechanosensitive calcium-permeable channels.

### 2.2. Molecular Dynamics Reveal Differential Lipid Accessibility for F22, R343, and H236

**Figure 3:**
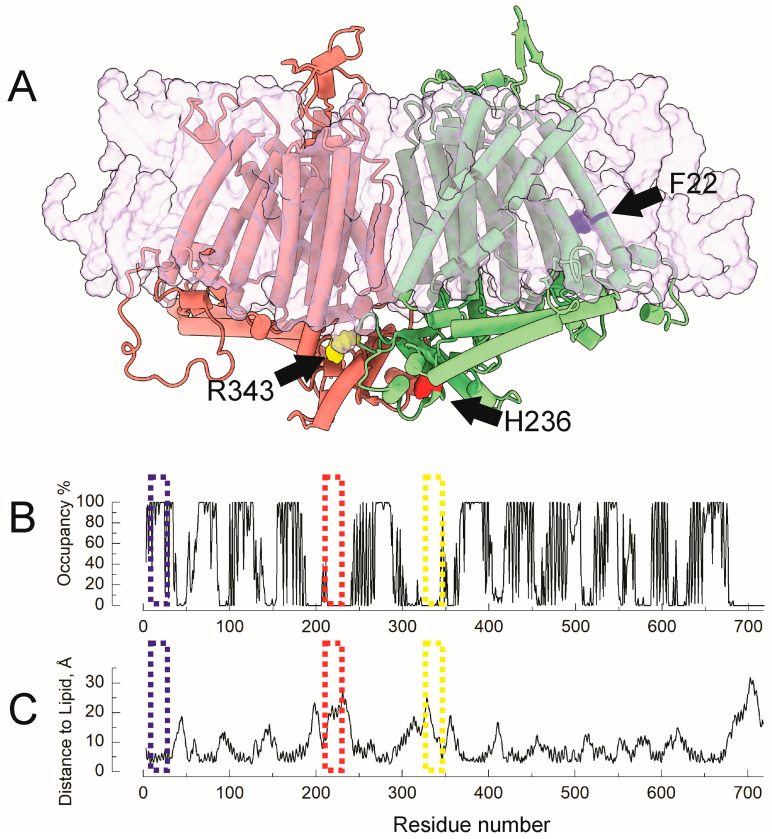
Lipid proximity and membrane exposure of key residues F22 and H236 in OSCA1.2. (**A**) Structural model of the transmembrane protein complex shown in cartoon representation with a semi-transparent surface overlay for the lipid bilayer. Individual subunits are colored green and salmon. Residues F22 (blue), H236 (red), and R343 (yellow), whose crosslinking impaired channel function when substituted with BzF (Figure 2), are shown in van der Waals representations and highlighted with black arrows. (**B**) Lipid interaction occupancy profile for each residue over the course of the molecular simulation, with a cutoff distance for a positive interaction of 5Å. (**C**) Average distance (in Å) from each residue to any lipid molecule throughout the simulation. Residues F22, H236, and R343 are enclosed by blue, red, and yellow boxes, respectively.

Mechanosensitive ion channels can be directly opened by mechanical forces transmitted through the lipid membrane—a phenomenon known as the “force-from-lipids” [21,22]. To assess whether specific residues implicated in OSCA1.2 gating interact directly with membrane lipids, we performed all-atom molecular dynamics (MD) simulations of the OSCA1.2 channel embedded in a POPC lipid bilayer in its closed conformation (PDB: 8XW3). We focused on the membrane/protein interaction of three functionally relevant residues: F22, R343, and H236. Figure 3A illustrates their positions within the dimeric structure: F22 (blue) is located deep in the transmembrane core facing TM6 and TM9; H236 (red) is situated at the cytosolic base of the dimerization interface, distant from the lipid bilayer; and R343 (yellow) lies at the intracellular loop between TM4 and TM5, near the membrane interface. To quantify lipid interactions, we measured both lipid occupancy (fraction of time lipids are within 5Å of a given residue) and distance to nearest lipid atoms over the 1 μs trajectory. Figure 3B shows the lipid occupancy per residue during the simulation, with a threshold of positive interaction of 5Å. In Figure 3B, F22 (blue box) shows consistently high lipid occupancy, indicating persistent contact with surrounding lipids. H236 (red box) remains largely devoid of lipid contact throughout the simulation and R343 (yellow box) exhibits moderate lipid interaction, suggesting partial exposure. Figure 3C shows corresponding average lipid distances: F22 remains closely associated with membrane lipids, while H236 maintains a greater separation. R343 falls in between, displaying fluctuating proximity to lipids. Together, these data point to a mechanistic distinction among the three residues. F22 is located at the membrane–protein interface and is well positioned to participate in lipid-mediated force transduction. R343 resides within the transmembrane core and may serve as a structural relay between membrane deformation and intracellular gating machinery. In contrast, H236 is located in the intracellular domain and likely contributes to gating through internal conformational changes, independent of direct lipid interactions.

### 2.3. Contact and Correlation Analyses Suggest Dual Allosteric Pathways to Y519 in OSCA1

**Figure 4:**
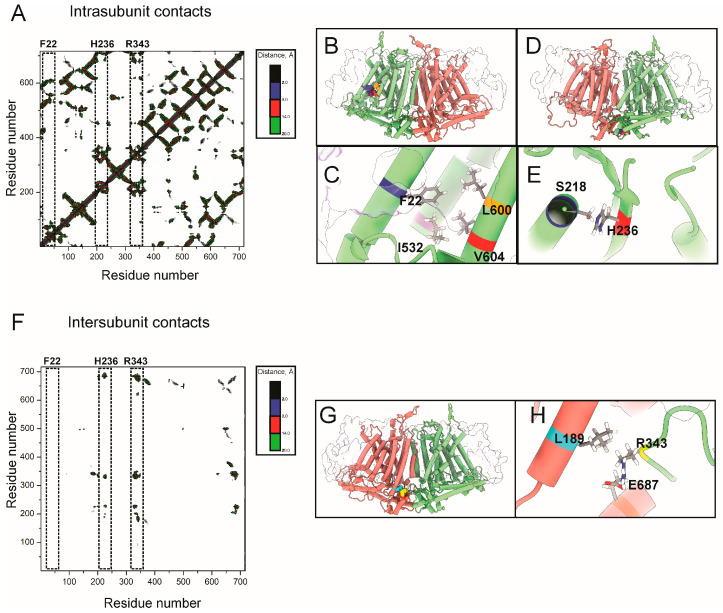
Intra- and inter-subunit contacts analysis reveals structural coupling between N-terminal helix, activation gate, and dimer interface in OSCA1.2. (**A**) Intra-subunit distance matrix for a single OSCA1.2 protomer, highlighting close-contact residue pairs. F22, H236, and R343 are indicated with dashed boxes. Contact distances are color-coded from 2 Å (blue) to 20 Å (green). (**B**,**C**) Structural models showing OSCA1.2 as a homodimer (salmon and green), with contact residues F22 and H236 highlighted. R343 was omitted for not showing significant intra-subunit contacts (see main text) (**D**) Close-up of F22 forming a hydrophobic cluster with I532, V604, and L600 near TM6, suggesting a stabilizing role near the proposed activation gate (Y519 lies nearby). (**E**) H236 lies adjacent to S218 on TM3, suggesting a stabilizing interaction potentially involved in gating. (**F**) Inter-subunit distance map showing residue contacts between opposing protomers. Interfacial contacts for F22, H236, and R343 are indicated with dashed boxes. (**G**) Structural models showing OSCA1.2 as a homodimer (salmon and green), with contact residue R343 highlighted. F22 and H236 were omitted for not showing significant inter-subunit contact (see main text). (**H**) Close-up of the inter-subunit interaction cluster centered on R343 (chain A), L189, and E687 (chain B).

Contact maps are valuable tools for identifying energy linkage between distant domains in both soluble and membrane proteins [23,24]. To dissect the structural basis of mechanogating in OSCA1.2, we combined contact mapping and dynamic cross-correlation (DCC) analysis to explore how key residues interact within and across subunits. The contact maps revealed two distinct intra-subunit interaction networks (Figure 4A). The first centers on F22, located at the N-terminal region of TM0, which forms stable hydrophobic contacts with pore-adjacent residues I532, L600, and V604 (Figure 4B,C). This cluster situates F22 potentially coupled to Y519 on TM6, a conserved residue proposed to form part of the activation gate (see also Figure 3). A second intra-subunit interaction hub involves H236, located on the IL2β2 loop, which engages in defined contact with S218 within the same subunit (Figure 4D,E). R343 also showed several intra-subunit contact hotspots, but its side chain orientations make it unlikely to become a crosslinking partner after UV exposure. Inter-subunit contact analysis (Figure 4F) shows no contact for F22 and only a few hotspots for H236. However, the side chain orientation of H236 makes crosslinking unlikely, consistent with its cytosolic exposure. Our data indicates that R343 exhibits partial lipid exposure and intermediate proximity to the membrane (Figure 3). Additionally, R343 engages in partial inter-subunit contact across the dimer interface with E687 and L189 (Figure 4G,H). This configuration suggests that R343 is positioned to constrain motion at the dimer core in the resting state.

**Figure 5:**
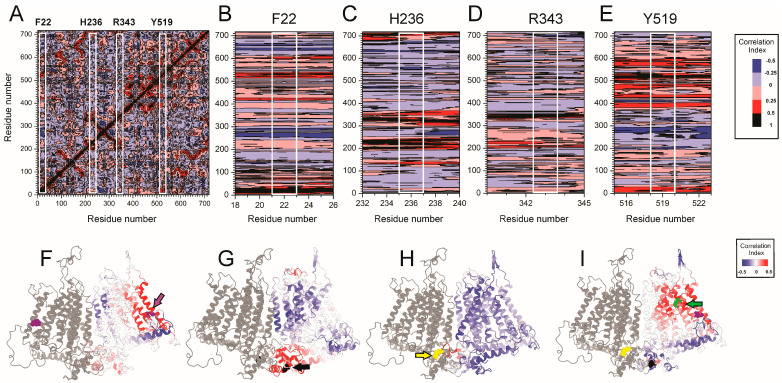
Dynamic cross-correlation analysis reveals distinct allosteric interaction networks involving F22, H236, R343, and Y519 in OSCA1.2. (**A**) Global dynamic cross-correlation matrix (DCCM) from 1 μs molecular dynamics simulations of OSCA1.2. Axes represent residue indices. Color scale indicates the magnitude and direction of correlated motions. White boxes mark the positions of F22, H236, R343, and Y519. Residue-specific cross-correlation profiles centered on F22 (**B**), H236 (**C**), R343 (**D**), and Y519 (**E**). Each row shows the correlation of the focal residue (white lines) with all others. (**F**–**I**) Structural representations of OSCA1.2 dimers highlighting residues and regions dynamically correlated with the focal residues. One protomer is shown in gray; the other is colored by correlation intensity (blue = anticorrelated, red = positively correlated). Key residues are pointed by arrows and shown in van der Waals representation: F22 (purple), H236 (black), R343 (yellow), and Y519 (green). Color scales apply to panels (**F**–**I**), ranging from –0.5 (anticorrelated, blue) to >0.5 (correlated, red).

To explore coordinated motions network that may underlie mechanogating in OSCA1.2, we performed dynamic cross-correlation (DCC) analysis over the 1 μs molecular dynamics trajectory [25,26]. Figure 5A presents a global DCC matrix where each pixel represents the correlation coefficient between residue pairs, ranging from positively correlated (red) to anticorrelated (blue). Closed state-trapping residues F22, H236, R343, and the channel activation gate Y519 are enclosed in white boxes to guide interpretation. Residue-specific correlation profiles (Figure 5B–D) reveal distinct motion signatures. F22 (Figure 5B), located in TM0, displays localized positive correlations with IL1H1 (residues ~70–75), TM4 (~420–425), and TM6 (~520–525), consistent with its role in membrane-coupled gating and proximity to the pore-lining Y519. H236 (Figure 5C), situated in the IL2β2 loop, exhibits broader dynamic coupling across cytosolic elements, including regions 130–140, H2β3–IL2H4 (320–338; 344–348), and the distal C-terminal tail (700–714), suggesting a role in long-range allosteric signaling. R343 (Figure 5D) shows strong anti-correlations with transmembrane and intracellular regions, including TM1, TM3, and IL4, indicating that it may constrain conformational transitions by stabilizing the dimer interface. Figure 5E shows the correlation landscape for Y519, a residue within TM6 proposed to act as the channel’s activation gate. Notably, Y519 exhibits positive dynamic coupling with both F22 and R236, while there is a small correlation with H343. Structural projections of these correlation networks (Figure 5F–I) map the extent and spatial organization of these allosteric interactions. F22-linked motions (Figure 5F, purple) cluster in the membrane core, while H236 correlations (Figure 5G, black) span the cytosolic interface. R343 (Figure 5H, yellow) exhibits interfacial coupling within the dimer, consistent with its trapping function. Y519 (Figure 5I, green) integrates signals from all three residues, forming the nexus of an allosteric relay system. Together, these results suggest a triad-based allosteric model for OSCA1.2 gating: F22 transduces force from the membrane, H236 coordinates structural shifts across cytosolic elements, and R343 stabilizes the closed state at the dimer interface. These dynamic pathways converge on Y519, enabling the channel to integrate mechanical and structural cues for efficient gating.

## 3. Discussion

Advances in structural prediction tools like AlphaFold and high-resolution techniques such as Cryo-EM have transformed our understanding of ion channel architecture [27]. However, elucidating the mechanisms by which mechanical forces gate these proteins still requires experimental strategies that capture conformational dynamics in a functional context. Mechanosensitive ion channels such as OSCA1.2 exemplify this challenge. Unlike voltage-gated or ligand-gated channels, which often rely on conserved structural motifs like S4 voltage-sensing domains or canonical pore helices [28], mechanosensitive ion channels display significant sequence and structural diversity and are primarily defined by their sensitivity to mechanical stimuli [2]. OSCA1.2 is gated by hyperosmotic stress and exhibits a unique proteo-lipidic pore architecture in which lipid molecules are integral structural components. This contrasts with tension-sensitive bacterial channels like MscL that respond directly to bilayer stretching via the displacement of pore-lining helices [21,29,30] and resembling more complex gating strategies seen in TMEM16 [31], Piezo, and TRAAK channels [32,33,34]. OSCA1.2 appears to couple lipid interactions with internal conformational signaling into the channel permeation pathway, whose status is proposed to be controlled by the orientation of Y519.

Using BzF-mediated photo-crosslinking, we identified three functionally important residues—F22, H236, and R343—as contributors to the gating process. The immobilization of F22, H236, and R343 via UV light irreversibly prevented channel reopening in response to a second hyperosmotic stimulus (Figure 2), indicating that conformational flexibility at these positions is essential for reactivation. F22 resides in TM0, oriented toward TM6, and maintains persistent interactions with membrane lipids (Figure 3), consistent with a role in lipid-mediated mechanogating [35,36]. H236, by contrast, is positioned in the IL2β2 loop at the base of the lipid cleft—distant from the membrane core. Located in the TM4–TM5 loop, R343 displays partial lipid exposure (Figure 3) and forms inter-subunit contacts (Figure 4), suggesting that it functions as a closed-state latch that stabilizes the dimer interface [35,37]. Molecular simulations helped contextualize these functionally sensitive residues. F22, positioned in TM0 and oriented toward TM6, maintained high lipid occupancy and remained embedded within the membrane throughout the trajectory (Figure 3). Considering the latter, it seems plausible that F22 plays a lipid-mediated mechanogating role, acting as a primary transduce linking tension changes at the lipid bilayer to TM6 and the pore-lining region. Interestingly, the F22BzF variant experienced a second pulse potentiation in control experiments. It is tempting to speculate about how the increase in size and hydrophobicity may lead the channel into a preactivated state that increases the channel open probability during the second pulse, but additional experiments are required to test this proposal. In contrast, H236—embedded in the IL2β2 loop at the base of the lipid cleft—exhibited negligible lipid contact but remained close enough to S218 to form stable intra-subunit interactions (Figure 4), suggesting a role in cytosolic allosteric communication rather than membrane sensing [38]. Arginine 343, the third residue showing UV-related effects, resides near H236 but engages distinct inter-subunit partners (e.g., E687 and L189) and appears to constrain the channel in the closed state, possibly serving as a mechanical brake that must be released for gating to proceed. Despite expectations of functional sensitivity due to its closeness to the channel pore, UV exposure had no effect on OSCA1.2 F389BzF channels (Supplementary Materials Figures S1 and S2). This suggests that local structural plasticity can buffer the functional impact of crosslinking even in spatially constrained regions. These examples illustrate that gating sensitivity to immobilization is governed not merely by residue proximity to critical domains, but by their dynamic context within the channel structure. Dynamic cross-correlation analysis resolves the functional relationships among key gating residues in OSCA1.2. The global DCC matrix shows that Y519 exhibits strong positive correlation with F22, consistent with their physical proximity and potential mechanical linkage via TM0–TM6 interactions (Figure 5A,B,F). H236, although spatially distant from Y519, shows measurable long-range correlation (Figure 5C,G), suggesting that it transduces mechanical information through a relay of dynamically coupled residues, including IL2H2—a putative hinge connecting the cytosolic interface to the TM6 gate [13,14]. R343 exhibits dynamic anti-correlated movements with most channel domains, including Y519, with stronger correlations observed in segments lining the dimer interface (Figure 5D,H). These findings make plausible a triadic allosteric communication pathway working together during OSCA1.2 channel gating. In this model, F22 transmits mechanical force from the lipid bilayer directly to TM6 via membrane-embedded rearrangements [35,36]. H236, residing in the IL2β2 loop, participates in an internal cytosolic relay that modulates gating through inter-domain coordination, consistent with its proposed role as a mechanosensitive node within the OSCA/TMEM63 family [37,39]. Meanwhile, R343, situated at the dimer interface, functions as a closed-state stabilizer; it must be displaced to allow the channel to open, contributing to a tension-sensitive latch mechanism [35,37,39]. These pathways may converge dynamically and structurally on Y519, consistent with its central role at the activation gate [13]. The functional impact of restricting the motion of F22, H236, and R343 illustrates how lipid sensitivity, cytosolic communication, and dimer interface mechanics integrate into a unified gating mechanism. This model aligns with evidence from TMEM63a homologs, which retain mechanosensitivity as monomers but require elevated pressure for activation, suggesting that dimeric mobility reduces the activation threshold in OSCA1.2 [40]. Future studies combining force-probe MD simulations, targeted mutagenesis, and tension-resolved electrophysiology are essential to elucidate the temporal order of these conformational transitions and to determine whether triad-based allosteric control represents a conserved gating strategy across the OSCA/TMEM63 superfamily.

## 4. Materials and Methods

### 4.1. Molecular Biology

The pcDNA3.1 expression vector encoding the wild-type OSCA1.2 channel from *Arabidopsis thaliana* was used as the backbone for generating mutant constructs with Amber (TAG) stop codons allowing the incorporation of the unnatural amino acid (UAA) p-benzoyl-L-phenylalanine (BzF). OSCA1.2 plasmid (Addgene plasmid # 136592) was a gift from Dr. Ardem Patapoutian (Scripps Institute, San Diego, CA, USA). Site-directed mutagenesis was performed by Synbiotech (Monmouth Junction, NJ, USA) and the sequences of plasmids were verified by double-stranded sequencing.

### 4.2. Cell Culture, Transfection, and UAA Incorporation

HEK293 *Piezo1*^−/−^ cells (a gift from Dr. Eduardo Perozo, University of Chicago, Chicago, IL, USA) were kept in MEM supplemented with 10% fetal bovine serum at 37 °C with 5% CO_2_. Cells were transfected using Xfect (Takara Bio USA, Inc., San Jose, CA, USA) with plasmids encoding OSCA1.2 constructs and vectors for the orthogonal BzF tRNA and aminoacyl-tRNA synthetase pair. pIRE4-BzF plasmid (Addgene plasmid # 155342) was a gift from Dr. Irene Coin (Leipzig University, Leipzig, Germany). The mass ratio for transfection was 3:1 (OSCA1.2: tRNA and aminoacyl-tRNA synthetase pair.). Five hours after transfection, the medium was replaced with MEM supplemented with 5 mM BzF (CHEM-IMPEX INTERNATIONAL, Inc., Wood Dale, IL, USA), prepared by dissolving BzF in 1 M HCl, adjusting the pH to 7.3 with NaOH, and filter-sterilizing (0.22 μm PVDF filter). Cells were incubated with BzF for 24–48 h before experiments.

### 4.3. Calcium Imaging

Transfected HEK293 cells were loaded with 4 μM Fluo-4 AM (Thermo Fisher, Waltham, MA, USA) in Hank’s Balanced Salt Solution (HBSS) containing 1 mM CaCl_2_ for 30 min at 37 °C. HEK293 *Piezo1*^−/−^ cells were cultured in Minimum Essential Medium (MEM) supplemented with 10% fetal bovine serum (FBS) at 37 °C in a 5% CO_2_ atmosphere. A two-pulse stimulation protocol was employed. Fluorescence was first recorded under baseline conditions (C), followed by a ~40 s hyperosmotic stimulus with 0.6 M NaCl (P1). After recovery in isotonic HBSS, a second identical pulse (P2) was applied. In UV-related groups, a 365 nm light pulse (30–40 s) was delivered during the recovery phase between P1 and P2 using a Mic-LED-365 light source (Prizmatix, Holon, Israel, 200 mW) through the optical path of an Olympus IX70 microscope (Olympus Corp., Tokyo, Japan) equipped with a 20× 0.6 NA PlanApo objective. Fluo-4 excitation was provided by a UHP-T-450-EP high-power LED (450 ± 6 nm), and emission was recorded using an OptiMOS camera (QImaging, Surrey, BC, Canada). Images were acquired at 1 Hz using Micro-Manager 2.0 software [41]. Fluorescence signals were quantified and reported as (F − F_o_)/F_max_, where F is the fluorescence intensity at time t, F_o_ is the baseline fluorescence before the hyperosmotic pulse, and F_max_ is the maximum fluorescence observed during P1. This normalization constrains fluorescence values between 0 and 1 for all experiments except F22BzF, which exhibits a second pulse potentiation rendering values larger than 1 for P2 in control experiments. Signal decay over the entire experiment was corrected by fitting a single-exponential decay function to the time series, as previously described [42]. Each biological replicate (n) corresponds to the average of at least 10 representative cells selected from the same field of view. These replicate means were used to compare P2/P1 ratios between the control and UV-treated groups, with statistical analysis performed using the Student’s *t*-test in GraphPad (version 10.5.0).

### 4.4. Molecular Simulations

The starting structure for molecular dynamics (MD) simulations was based on the cryo-electron microscopy (cryo-EM) structure of the *Arabidopsis thaliana* OSCA 1.2 protein (PDB ID: 8XW3), resolved in its expanded conformation at a 3.63 Å resolution [37]. Missing residues and loops in the protein structure were modeled using the Prime module Missing residues and loops in the protein structure were modeled using the Prime module in the Schrödinger Maestro Suite (version 2022-04, Schrödinger, LLC, New York, NY, USA). Regions lacking electron density—typically flexible loops or terminal residues—were identified using the Protein Preparation Wizard within the same suite. These missing segments were modeled to obtain a structurally complete and physically realistic protein model for further simulations and analyses. Loop modeling was performed using the knowledge-based and energy-based approach implemented in Prime. Specifically, the loop prediction tool was used to reconstruct segments with absent coordinates, with the default settings employed for sampling loop conformations. Prime generated an ensemble of candidate conformations, from which the lowest energy conformation based on the OPLS4 force field [43] was selected. The full structure underwent global energy minimization to ensure stability and eliminate any unfavorable interactions introduced during modeling. This completed and refined model was used as the input for subsequent molecular docking and molecular dynamics simulations. The refined OSCA1.2 model was embedded in a palmitoyl-oleoyl-phosphatidylcholine (POPC) lipid bilayer using CHARMM-GUI [44]. The membrane-integrated assembly was solvated in a TIP3P water box with 150 mM NaCl to approximate physiological ionic strength. All titratable residues were assigned their standard protonation states at pH 7.0. System preparation resulted in a membrane–protein–solvent assembly suitable for long-timescale MD simulations. Computational simulations were conducted using NAMD3 [45] leveraging GPU acceleration technology for improved processing efficiency, employing the CHARMM36m [46] force field for protein components and CHARMM36 [47] for lipid molecules. Long-range electrostatic forces were computed via the particle mesh Ewald (PME) approach with a 1.2 nm cutoff distance [48]. An identical cutoff was implemented for van der Waals interactions, incorporating a switching function beginning at 1.0 nm. Hydrogen-containing covalent bonds were maintained rigid using the SHAKE constraint algorithm, permitting a 2 fs integration timestep. Initial system optimization employed the steepest descent minimization method, succeeded by a staged equilibration procedure in both NVT and NPT statistical ensembles. Following energy optimization, the assemblies underwent NVT equilibration (0.25 ns) and subsequent NPT equilibration (4.6 ns) through the gradual reduction of restraining forces on lipids and proteins, plus an additional 150 ns of unrestrained NPT equilibration. Production-phase simulations operated under NPT conditions, maintaining constant temperature at 273 K for 500 ns and at 323 K for an additional 500 ns per system using Langevin temperature control, totaling 1 μs per system. Pressure regulation at 1 bar was achieved through the Langevin piston pressure control method with semi-isotropic coupling [49]. Simulation visualization and data analysis were performed using VMD [50] and Chimera X 1.9 [51] software packages.

### 4.5. Dynamic Cross Correlation Analyses

To evaluate the effect of temperature on the protein dynamics, dynamical cross-correlation maps [25,26] were calculated using the Bio3D package in R 4.0 [52]. Dynamic cross-correlation matrices with coefficients

*C*_*ij*_ = 〈Δ*r_i_*·Δ*r_j_*〉〈Δ*r_i_*_2_〉〈Δ*r_j_*_2_〉

were determined from the positions of the main chain C*α* atoms in amino acids *i* and *j* with positions *r*_*i*_ and *r*_*j*_. Δ*r*_*i*_ and Δ*r*_*j*_ determine the displacement of the *i*th C*α* from its mean position over the entire trajectory.

## Data Availability

All datasets, reagents, and materials are available upon request.

## Supplementary Materials

The supporting information can be downloaded at https://www.mdpi.com/article/10.3390/ijms26157121/s1.
