# Supplementary material for: Unnatural Amino Acid Photo-Crosslinking Sheds Light on Gating of the Mechanosensitive Ion Channel OSCA1.2
Source: Int J Mol Sci. 2025 Jul 23;26(15):7121. doi: 10.3390/ijms26157121 (PMC12346119; doi:10.3390/ijms26157121)
Supplement: Supplementary file 1 [file ijms-26-07121-s001.zip › ijms-3669991-supplementary.pdf]

International Journal of
Molecular Sciences

MDPI

Supplementary Figure S1

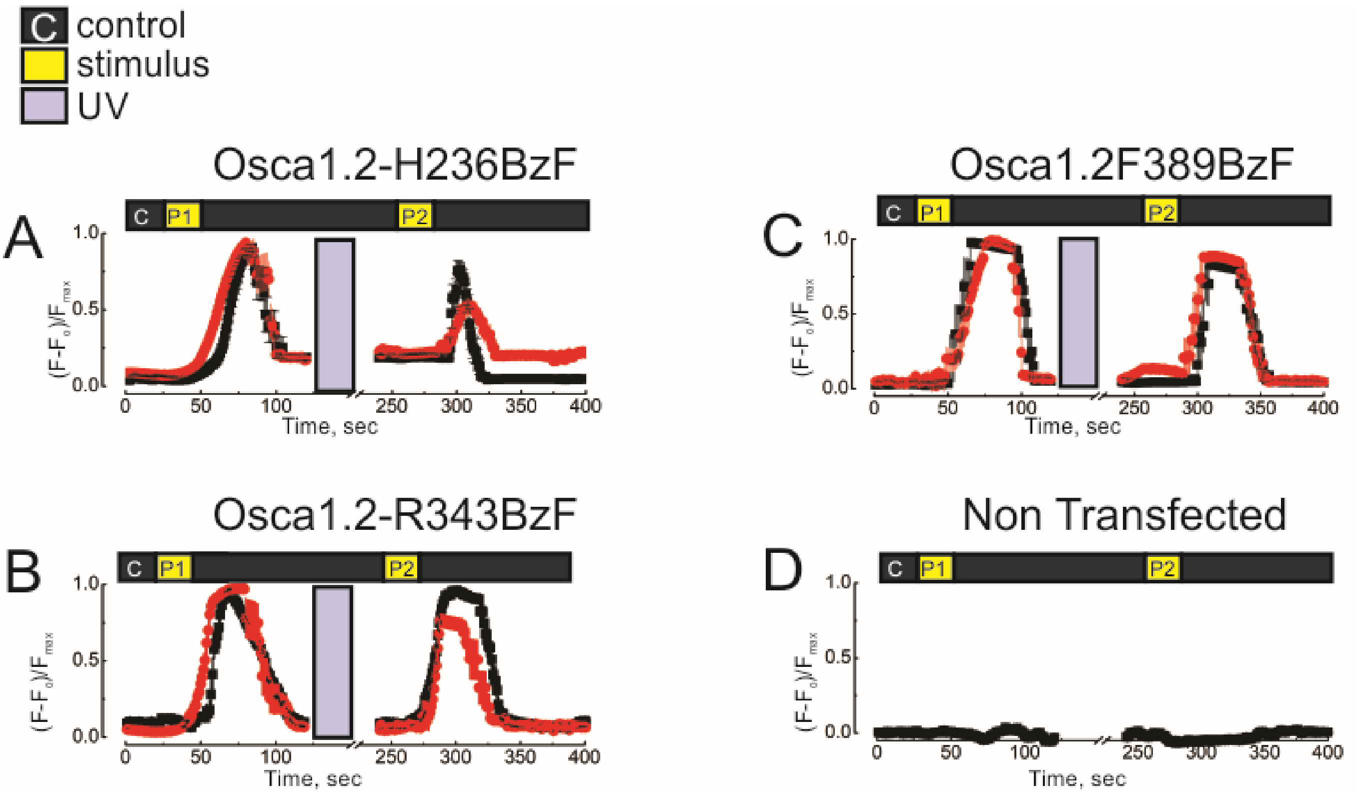

Supplementary Figure S1. Functional responses of Osca1.2 BzF variants to repeated mechanical stimulation and UV exposure.

(A–C) Average of normalized fluorescence traces (F-Fo/Fmax) over time for HEK293T *Piezo1*$^{-/-}$ cells expressing Osca1.2 with site-specific BzF incorporation at positions H236 (A), R343 (B), and F389 (C). Black traces represent the average of control experiments where no UV is applied during the recovery phase between P1 and P2. Red traces represent the average of experiments where cells are exposed to UV during the interpulse period. (purple box). (D) Non-transfected cells show no response under identical conditions. Control (C), stimulus (yellow boxes), and UV exposure (purple shading) periods are indicated above each panel.

Supplementary Figure S2

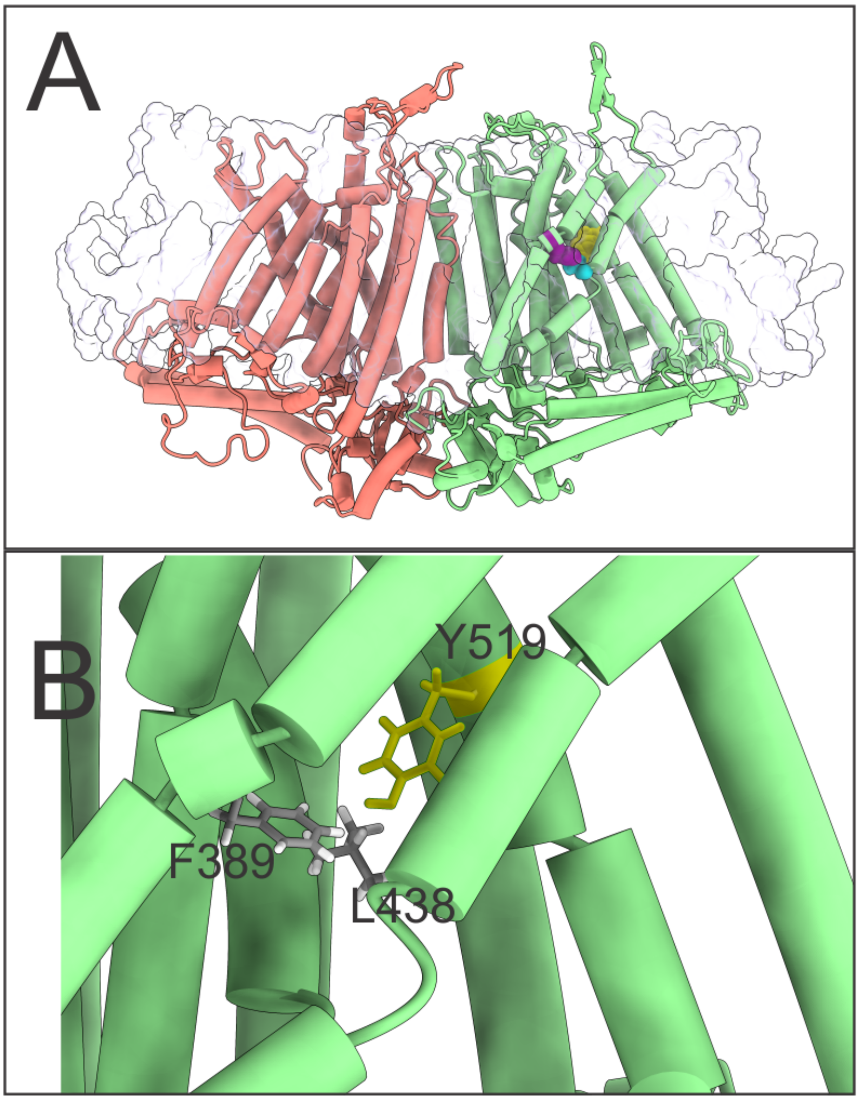

Supplementary Figure S2. Structural features at the Y519 activation gate.

(A) Overall structure of the heterodimeric protein complex shown in cartoon representation, with individual subunits colored light red and green. Residues F389, L438, and Y519 are colored in purple, yellow and cyan. (B) Zoomed-in view of the activation gate region showing residues F389, L438, and Y519. Y519 is shown in yellow, with adjacent residues F389 and L438 are depicted in gray.
